# The effects of dithiaden on nitric oxide production by RAW 264.7 cells

**DOI:** 10.2478/v10102-010-0044-9

**Published:** 2010-11

**Authors:** Jana Králová, Michaela Pekarová, Katarína Drábiková, Viera Jančinová, Radomír Nosáľ, Milan Číž, Antonín Lojek

**Affiliations:** 1 Institute of Biophysics, Academy of Sciences of the Czech Republic, Brno, Czech Republic; 2 Institute of Experimental Pharmacology, Slovak Academy of Sciences, Bratislava, Slovak Republic

**Keywords:** dithiaden, nitric oxide, RAW 264.7 cells, lipopolysaccharide, nitrites, inducible nitric oxide synthase

## Abstract

As reported in our previous studies, dithiaden (an antagonist of histamine H_1_-receptor, used clinically as an anti-allergic or anti-emetic drug) in a concentration range of 5×10^−5^–10^−4^ M decreased the production of reactive oxygen species by phagocytes. In this study we investigated the influence of dithiaden on nitric oxide (NO) production by LPS-stimulated macrophages.

The cell viability in the presence of 10^−4^–5×10^−5^ M dithiaden was evaluated by an ATP-test. RAW 264.7 cells (2.5×10^6^/well) were preincubated with dithiaden for 60 mins and subsequently stimulated with 0.1 µg/ml of bacterial lipopolysaccharide. After incubating for 24 hours the NO production was determined spectrophotometrically using Griess reaction as a concentration of nitrites (the end product of NO metabolism) accumulated in the cell supernatants. The expression of inducible nitric oxide synthase (iNOS) in cell-lysates was evaluated using Western blot analysis. Scavenging properties of dithiaden against NO were evaluated amperometrically.

Our data demonstrate that dithiaden in the concentration of 5×10^−5^ M (approved by ATP test as non toxic) caused a significant decrease in the accumulation of nitrites, and in addition, this decline was followed by a marked reduction of iNOS protein expression. Amperometrical analysis did not show any scavenging properties of dithiaden against NO.

From this data it can be suggested that the inhibition effect of dithiaden on macrophage NO production is caused exclusively by the suppression of iNOS protein expression.

**Abbreviations**
ATP: adenosine triphosphate
DMEM: Dulbecco's Modified Eagle's Medium
FBS: fetal bovine serum
iNOS: inducible nitric oxide synthase
LPS: lipopolysaccharide
NO: nitric oxide
PBS: phosphate buffer salt
SDS: sodium dodecyl sulfate

## Introduction

H_1_-antihistamine dithiaden (4[3-dimethylamino (propylidiene)]-4,9dihydrothieno-(2,3b)benzo-[e]thiepin) is a member of the 1^st^ class of histamine H_1_-receptor blockers and is used clinically as an anti-allergic or anti-emetic drug (De Vos, 1999). The suppresive effects of this group of drugs on histamine secretion from mast cells and basophils are well known. Experiments suggested that the chemical structure of H_1_-antihistamines, namely positively charged lipophilic molecules, allows them to associate with the cell membrane. Moreover, they are able to inhibit the activity of calcium-dependent enzymes, affect the calcium mobilization and the discharge of intracellular calcium stores as being responsible for various effects including histamine secretion, eicosanoids or reactive oxygen metabolites generation (Church, 2001; Lullmann *et al*., 1980). The production of reactive oxygen and nitrogen species by phagocytes belongs to the important microbicidal mechanisms of fight against pathogenic microorganisms, bacterias, tumor cells etc., in the process of inflammation. Normal inflammatory responses are self-limited by a process that involves the downregulations of pro-inflammatory proteins and the upregulations of anti-inflammatory proteins (Lawrence *et al*., 2002). But chronic inflammation accompanied by a rise in oxidative stress with an overproduction of reactive oxygen and nitrogen species could contribute to the pathogenesis of many inflammatory diseases, such as bronchitis or rheumatoid arthritis. Whereas the effect of dithiaden on the production of reactive oxygen species is relatively well described (Kralova *et al*., 2006; Nosal *et al*., 2002; Nosal *et al*., 2006), the information about the role of dithiaden on reactive nitrogen species generation is insufficient. Therefore, the present study investigated the effects of dithiaden on nitric oxide (NO) production by murine macrophages like cells RAW 264.7 stimulated by lipopolysaccharide and also tries to detect the direct scavenging properties of dithiaden against NO in a cell free system.

## Materials and methods

### Materials

Dithiaden (Zentiva, Czech Rep.) was dissolved in distilled water and the stock solution 3×10^−3^M was stored in −20°C. Lipopolysaccharide (LPS) from *Escherichia coli* serotype 0111:B4 (Sigma-Aldrich, USA) was dissolved in PBS and the stock solution 1mg/ml was stored in −20°C. Other chemicals were purchased from local distributors.

### RAW 264.7 cells

A murine leukaemic macrophage-like RAW 264.7 cells (ATCC, USA) were grown in plastic culture flasks in Dulbecco's Modified Eagle's Medium (DMEM) supplemented with heat-inactivated 10% fetal bovine serum (FBS), gentamycin, glucose and NaHCO_3_ in a CO_2_ incubator (5% CO_2_ and 95% of air humidity) at 37°C. Cells were seeded at an initial density of 2.5×10^6^ cells/well in a 6-well tissue culture plates and preincubated with dithiaden for 60 min. Cells were then stimulated with LPS (0.1 µg/ml) and incubated 24 h. Non-treated cells were used as the control. Cell supernatants were used for the determination of nitrite concentration, cells were used for the measurement of ATP content and iNOS protein expression.

### ATP test of cell viability

The viability of RAW 264.7 cells was tested by the commercial ATP cellular kit (Biothema, Sweden). Cells were incubated for 24 hours with 10^−4^ and 5×10^−5^ M dithiaden, supernatant was removed and cells were lyzed by Somatic cell ATP releasing reagent (Sigma Aldrich, USA). The volume of 50 µl of lyzate were mixed with a 20 µl ATP reagent SL containing D-luciferin, luciferase and stabilizers. Intracellular ATP contents were determined luminometrically using luminometer Orion II. (Berthold Detection Systems GmbH, Germany).

### Determination of nitrite production by cells

Detection of accumulated nitrites (NO_2_^−^) in the cell supernatants was performed using the Griess reagent as described previously (Migliorini *et al*., 1991). 150 µl of the cell supernatant was incubated with 150 µl Griess reagent (Sigma-Aldrich, USA) for 15 min in a dark at room temperature and the absorbance was measured at 546 nm on a Spectra Rainbow UV/Vis microplate reader (SLT Tecan, Germany). The concentrations of nitrites were derived by regression analysis using serial dilutions of sodium nitrite as a standard.

### Determination of iNOS protein expression by cells

Cells were lysed with 1% SDS lysing buffer with the addition of 1% phenylmethanesulphonylfluoride. Protein volume in cell samples was determined using commercial BCA protein assay (Pierce, USA), proteins (22 µg) were separated by 7.5% SDS-PAGE and then transferred to a polyvinylidene difluoride membrane in a buffer containing Tris-glycine and 20% methanol. Protein was labeled using mouse antibody (1:1000) specific to iNOS (Becton – Dickinson, USA) and HRP-conjugated goat anti-mouse antibody (1:2000) (ECL™ Anti-mouse IgG, Amersham, Biosciences, USA), subsequently immunoreactive bands were visualised using ECL detection reagent (Detection reagents kit, Pierce, USA).

### Amperometrical detection of NO scavenging

The measurement of NO scavenging was performed amperometrically using ISO-NO Mark II NO meter (World Precision Instruments, Inc. USA). The measurement was practised using NO standard solutions prepared with pure NO gas. Preparation of 2 mM NO stock solution was described earlier (Feelisch and Kelm, 1991). 1 µl of NO stock solution was injected either into the 10 ml of PBS in a glass vial or into the 10 ml of PBS with the addition of 5×10^−5^ M dithiaden and the signal was measured for 10 min. Then the integrals of control curve and sample curve were calculated and the scavenging activity of the dithiaden was evaluated.

### Statistical evaluation

Data are expressed as the mean ± standard error of the mean (SEM) of at least 3 independent experiments, that were run in duplicates. Results were analysed by Student's two-tailed t-test using Statistica software (StatSoft, USA), values below 0.05 (*) were considered statistically significant.

## Results

Nitrite production was dependent on the activating state of RAW 264.7 cells. While only very low concentrations of nitrites (0.5–2 µM) were detected in non-stimulated cells, the cells stimulated with 0.1 µg/ml LPS generated 28.2 ± 2.52 µM (mean ± SEM) of nitrites. The value of nitrite accumulation after LPS-stimulation was used as a positive control.

**Figure 1:**
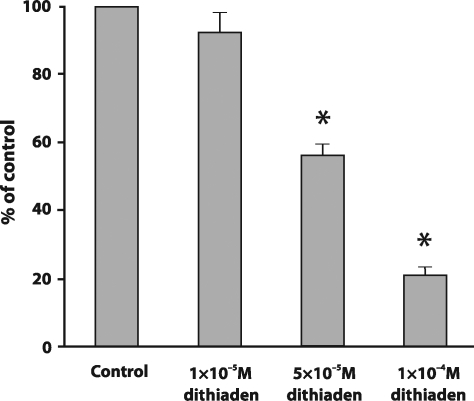
The effect of dithiaden on the concentration of nitrites in the supernatant collected from RAW 264.7 cells stimulated by 0.1 µM LPS after a 24 hour incubation period at 37°C in comparison with non-treated cells (control). Data are expressed as percentages of the control. Values represent mean ± SEM, n=3. Asterisks show significant differences as compared to control, *p<*0.05 was considered as statistically significant as compared to control.

Figure 1 demonstrates that the lowest concentration of dithiaden (10^−5^ M) did not exert ability to modulate nitrite production in cells stimulated with LPS. 5×10^−5^ M dithiaden significantly decreased the nitrite concentration (56.06 ± 3.34% of positive control level). Dithiaden in concentration 10^−4^ M also affected the nitrite accumulation (20.93 ± 2.19% of positive control level) in LPS-stimulated cells. However, this concentration of dithiaden also caused a significant decline in intracellular ATP concentrations (data not shown) and exerted a strong cytotoxic effect on RAW 264.7 after 24 hours of incubation. Therefore, the 5×10^−5^ M concentration of dithiaden which proved to be non toxic but still effective was used for the following experiments with cells.

**Figure 2:**
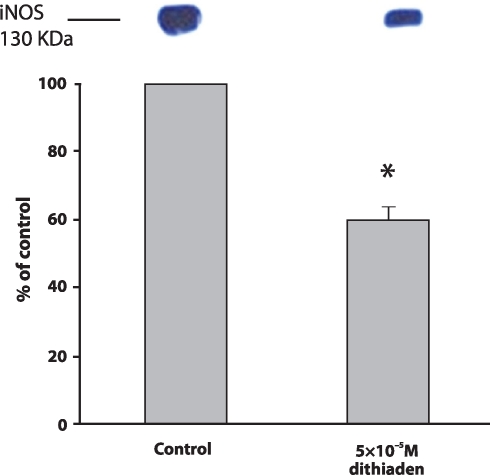
The expression of iNOS in the cell lysates from RAW 264.7 cells stimulated by 0.1 µM LPS after 24 hour incubation with 5×10^−5^ M dithiaden at 37°C in comparison with non-treated cells (control). Data are expressed as percentages of the control. Values represent mean ± SEM, n=3. Asterisks show significant differences as compared to control, *p<*0.05 was considered as statistically significant as compared to control.

The possibilty that the decrease in nitrite concentration was induced by a suppressioon of iNOS protein expression was verified in subsequent experiments. Figure 2 shows that 5×10^−5^ M dithiaden inhibited the iNOS protein levels in cell lysates to 59.88 ± 4.03% of positive control level (cells stimulated with LPS).

**Figure 3:**
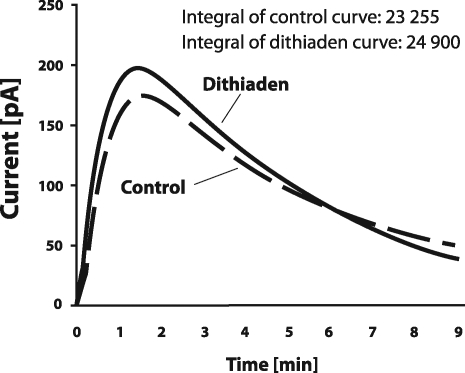
The effect of 5×10^−5^ M dithiaden against nitric oxide (NO) evaluated amperometrically. The electrochemical signal was induced by the addition of the NO stock solution as described in the text. Data are expressed as mean ± SEM, n = 3.

Dithiaden was further tested for scavenging NO by direct amperometric analysis using a carbon electrode selective for NO. Dithiaden in the 5×10^−5^ M concentration did not approve any scavenging properties against nitric oxide in PBS as shown in Figure 3.

## Discussion

Althought the number of recently published results (Jancinova *et al*., 2006; Kralova *et al*., 2006; Nosal *et al*., 2002; Nosal *et al*., 2005; Nosal *et al*., 2006) have demonstrated that dithiaden and other H_1_-antihistamines play a very important role in the regulation of reactive oxygen species production and also in the regulation of myeloperoxidase activity (Kralova *et al*., 2008), any information about the effect of H_1_-antihistamines on the generation of reactive nitrogen species is yet to be determined. We consequently investigated the role of dithiaden in the regulation of nitric oxide production in macrophages. Detection of iNOS protein expression and nitrite concentration are reliable methods for verifying NO production by cells. NO is generated by several types of cells in an L-arginine – pathway in the presence of nitric oxide synthases (NOS) (Moncada and Higgs, 1993; Palmer *et al*., 1987). iNOS is mainly expressed in macrophages in response to inflammatory stimuli, e.g. Gram-negative bacterial lipopolysaccharide or proinflammatory cytokines (IL-1 or TNF-α) (Stuehr and Marletta, 1985). We used this phenomenon for the *in vitro* experiments with a murine macrophage cell line RAW 264.7. We suggested that dithiaden (10^−4^ M and 5×10^−5^ M) caused a significant decrease in nitrite accumulation and also in iNOS protein expression in LPS-treated cells. However, only the 5×10^−5^ M concentration of dithiaden was non-toxic.

NO can be a double-edged sword. On one hand, NO is an important molecule involved in the regulation of many physiological and microbicidal processes. On the other hand, its overproduction is included in several chronic inflammatory diseases such as bronchitis, osteoarthritis or rheumatoid arthritis (McInnes *et al*., 1996). It is evident from our results, dithiaden does not directly scavenge nitric oxide generated in chemical system, this H_1_-antihistamine drug exerted significant inhibitory effects on the nitric oxide production by a murine macrophage cell line RAW 264.7. It can be concluded that this inhibition of nitric oxide production was caused by a decrease in iNOS expression.

We suggest that dithiaden by decreasing the nitric oxide production might contribute to the treatment of chronic inflammatory processes.
